# The Great Medical Temperance Movement

**Published:** 1891-06

**Authors:** 


### THE GREAT MEDICAL TEMPERANCE MOVEMENT.

So impressed is the JOURNAL with the importance of the movement, and so in sympathy with it, that a large portion of space in this number is devoted to the publication, in full, of the call for a Conference of Medical Men at Prohibition Park, Staten Island, N. Y., this summer. Two days—July 16 and 17, will be devoted to the temperance question.

The medical profession are at last awakening to a realizing sense of the magnitude of this great evil, and what is more to the point, to a recognition of the fact that *they have*, unwittingly, it is true, been a factor in its production, by the indiscreet, not to say, in some instances, reckless prescribing of alcohol—especially in chronic diseases.

It is now admitted as established that an induced habit may be transmitted from parent to child; and all know with what facility the habit of drink is induced when once begun. Hence, the physicians of the United States are called to meet in a general conference, to take stock, as it were, and determine to what extent *they* are responsible for the misery and woe entailed by drink, and what is more to the purpose,—their duty in the premises. Having seen and admitted their responsibility, in a measure, for the production of intemperance in this country, it is sought to be determined what shall be done about it; how to arrest the current; in what way to begin a reform moment. Prohibition, as a political measure, failed. The medical associations in convention have, as a general thing, turned a deaf ear to the appeals of the W. U. C. A. for co-operation in their efforts to suppress the liquor traffic and stop the evil of intemperance; the work of the Y. M. C. A. has met with ridicule, or at best a half-hearted second, and the outlook is indeed discouraging. But there are some good and brave men who are willing, for the sake of doing good, to brave popular opinion, and if necessary, incur the enmity of the whisky combine. Foremost amongst these is the great and good Davis; and it was he, who, recently, at Washington, we believe, gave the initial start, which has resulted in the call for this conference. God grant that it may be attended with success. Humanity cries out,—the wail of suffering women and children,—and the despair of hopeless drunkards,—call for *reform*.

If the medical profession have no influence in politics, and, as has been demonstrated in Texas, are powerless with legislatures in the demands for sanitary laws, whose necessity are clearly recognized,—if they cannot secure State aid in the arrest of intemperance, they can at least agree to make no more drunkards by sharpening the appetites of their patients for drink by prescribing it; they can, at least, put on record their unqualified condemnation of the sale of liquors; they can let the world know that *they* have long since abandoned the notion that alcohol is a "food, and conservative of tissue in fever,"—they can recommend that the evil effects of alcohol in the human system shall be taught in schools; they can ask, and easily secure the co-operation of all the women amongst whom they practice, in instilling into the youthful mind the great evils of alcohol.

In this connection Dr. W. A. Morris' paper, read at Waco, was most timely and appropriate. A copy of it will be sent to this conference, and the JOURNAL hopes to see it printed and circulated in every home in America.

Down with this modern moloch; down with this thrice terrible minataur,—to whose insatiable appetite ten thousand times "seven young maidens" and youths are yearly sacrificed. The monster of the Cretan labyrith was not a circumstance to this thrice devil—DRINK—which, unlike the miniataur, destroys body *and* soul.

