# Errata

**Published:** 1891-07

**Authors:** 


Errata.--In our last our compositors made us say, "The necessity for which 'are' clearly recognized.'' Beg pardon, you *know* we know better that that.

